# New occurrences of the bone-eating worm *Osedax* from Late Cretaceous marine reptiles and implications for its biogeography and diversification

**DOI:** 10.1098/rspb.2023.2830

**Published:** 2024-04-10

**Authors:** Sarah Jamison-Todd, Philip D. Mannion, Adrian G. Glover, Paul Upchurch

**Affiliations:** ^1^ Department of Earth Sciences, University College London, Gower Street, London, WC1E 6BT, UK; ^2^ Natural History Museum, Cromwell Road, London, SW7 5BD, UK

**Keywords:** bioerosion, biogeography, Cretaceous, marine reptiles, *Osedax*, Western Interior Seaway

## Abstract

The bone-eating worm *Osedax* is a speciose and globally distributed clade, primarily found on whale carcasses in marine environments. The earliest fossil evidence for *Osedax* borings was previously described in plesiosaur and sea turtle bones from the mid-Cretaceous of the United Kingdom, representing the only unequivocal pre-Oligocene occurrences. Confirming through CT scanning, we present new evidence of *Osedax* borings in three plesiosaur specimens and, for the first time, identify borings in two mosasaur specimens. All specimens are from the Late Cretaceous: one from the Cenomanian of the United Kingdom, two from the Campanian of the southeastern United States, and two from the Maastrichtian of Belgium. This extends the geographic range of *Osedax* in the Cretaceous to both sides of the northern Atlantic Ocean. The bones contain five borehole morphotypes, potentially created by different species of *Osedax*, with the Cenomanian specimen containing three morphotypes within a single tooth. This combined evidence of heightened species diversity by the Cenomanian and broad geographic range by the Campanian potentially indicates an earlier origin and diversification for this clade than previously hypothesized. Preservational biases indicate that *Osedax* was probably even more widely distributed and speciose in the Cretaceous than apparent in the fossil record.

## 1. Introduction

The bone-eating siboglinid annelid worm *Osedax* was described for the first time in 2004, associated with the remains of a dead whale (hereafter, a whalefall) discovered in Monterey Submarine Canyon, off the coast of California [1]. *Osedax* is mouthless and gutless; it digests lipids and/or collagen in bones with the aid of bacterial symbionts [2]. The general morphology of *Osedax* correlates to the borings that are left in the bone; each worm possesses palps that emerge from the borehole, while the trunk sits inside the aperture to the boring and the branching ovisac structure creates the chamber beneath the surface of the bone [1]. Concentrated research efforts in specific regions have resulted in the recognition of numerous species, with 18 species described from the Monterey Submarine Canyon alone [3]. There are currently 29 described species of *Osedax* [4] (World Register of Marine Species; https://marinespecies.org), forming a globally distributed marine clade that includes occurrences in the Atlantic, Indian, Pacific and Southern oceans, extending from 73.6°N in the Arctic [5] to 65.1°S in the Antarctic Peninsula [6] (Global Biodiversity Information Facility; https://www.gbif.org). The depth range of *Osedax* is also extensive, with living individuals discovered on vertebrate remains at depths of between 21 m [6] and 4208 m [7], showing its ability to adapt to a wide variety of marine environments, as long as there is bone and sufficient levels of oxygen present in those environments [8]. *Osedax* has been identified on both natural and implanted whalefalls, and has also been known to consume a variety of other bone substrates, including shark teeth [9] and turtle, turkey, cow, seal, pig and alligator bones [3]. One species (*Osedax japonicus*) has even been observed inhabiting soft tissue and blubber on a whale carcass off the coast of Japan [10]. Given that *Osedax* has been found wherever it has been sought thus far, on a wide range of vertebrate remains at various depths, it is likely to be much more speciose and widespread than is currently understood [6].

Initially, molecular clock divergence estimates placed the emergence of *Osedax* in either the Late Cretaceous or Late Eocene, depending on clock calibration [11]. These estimates required additional calibration, as it was not initially clear whether *Osedax* originated and diversified alongside the large cetaceans that are its preferred food source today, or whether it existed prior to the Cretaceous–Palaeogene mass extinction and was able to feed on marine reptile bones. More recent estimates place the origination of *Osedax* in the late Early Cretaceous at approximately 108 Ma [12], using the first instance of *Osedax* borings in the fossil record (greater than 100 Ma) as a calibration point [13].

Recognition of *Osedax* borings in the fossil record is based on unique features defined by the organism's morphology that have previously been described in detail in modern whale bone [14,15]. These borings consist of branching lobate chambers (diameter approx. 3 to approx. 10 mm) with a single entrance borehole (diameter approx. 1 mm) [15]. The size and branching morphology of the borings varies by species, but they are consistent enough in form to be recognizable as those of *Osedax* [15]. Borings resembling those created by *Osedax* are referred to the ichnogenus *Osspecus* [16]. Variation in borehole morphology within one species is dependent on variability in the type of bone [14,15]; individual species create consistent borehole morphologies within the same bone type that are distinct from those made by other species [15]. Therefore, multiple borehole morphologies in the same bone and bone type provide a measure of the diversity of species that has colonized that bone. Confirmation by CT scanning is required for confident identification of the borehole producer, because the external morphology of the borings cannot be unequivocally distinguished from boring and pitting produced by the many forms of bone weathering processes, other bioerosion, or bone pathologies. For example, clionaid sponges occasionally create borings in bone that can be mistaken for *Osspecus* unless the internal morphology of the borings is examined in detail [16–18], but no other studies have shown bioerosion in bone indistinguishable from *Osspecus* thus far.

*Osedax* borings in fossil whale bones are known from the Oligocene of Washington State, USA [19,20], the Pliocene of Italy [16], and tentatively described in the Oligocene of New Zealand [21]. The latter instance was not confirmed through CT scanning, but *Osspecus* is present in CT scans of the aforementioned examples, showing a broad distribution from the Mediterranean to the Pacific Northwest during the early evolutionary history of large marine cetaceans. *Osspecus* is also present in bird and fish bones in the Oligocene of Washington State [20,22]. There is one study that confirms the presence of *Osspecus* in the Mesozoic; these occurrences are in reworked Albian plesiosaur bones deposited in the Cenomanian Cambridge Greensand Formation and sea turtle bones from the Cenomanian Grey Chalk Subgroup of the United Kingdom [13]. CT scans reveal that these occurrences bear a strong resemblance to modern *Osedax* borings, suggesting that this organism was already highly specialized and occupied an ecological role and life mode similar to that of today [13]. In order to evaluate the spatiotemporal distribution of *Osedax* in the Mesozoic and further constrain the timing of its origination and diversification, new discoveries and further examination of the marine vertebrate fossil record are required.

In this study we describe five bioeroded specimens of plesiosaurs and mosasaurs from the Late Cretaceous of the United Kingdom, Belgium and the United States of America. We confirm the presence of *Osspecus* through CT scanning and three-dimensional reconstruction of the borings; this extends the geographical range of this taxon in the Late Cretaceous to both sides of the North Atlantic Ocean. Moreover, we describe multiple borehole morphologies and interpret this as evidence for a higher diversity of *Osedax* in the Late Cretaceous than previously recognized.

## 2. Material and methods

### (a) Data collection

The data and specimens for this study were sourced from collections containing marine reptile material mainly from the United States, mainland Europe, and the United Kingdom. Both Jurassic and Cretaceous marine reptile specimens from the following 13 collections were surveyed extensively for evidence of bioerosion: British Antarctic Survey, Cambridge, UK; Bristol Museum and Art Gallery, Bristol, UK; Cambridge Sedgwick Museum of Earth Sciences (CAMSM), Cambridge, UK; Field Museum of Natural History (FMNH), Chicago, USA; the Horniman Museum, London, UK; Muséum national d'Histoire naturelle, Paris, France; Natuurhistorisch Museum Maastricht, Maastricht, The Netherlands; the Natural History Museum, London (NHMUK); National Museum of Natural History, Smithsonian Institution, Washington DC, USA; Oertijdmuseum, Boxtel, The Netherlands; Oxford University Museum of Natural History, Oxford, UK; Institut royal des Sciences naturelles de Belgique (IRSNB), Brussels, Belgium; and Universitetet I Oslo Naturhistorisk museum, Oslo, Norway.

Plesiosaurs, ichthyosaurs, mosasaurs and thalattosuchian crocodylomorphs are the representative Mesozoic marine reptile taxa included in this study. Most of the material in these collections was sourced from the country in which the material is housed, but many collections also contained a small number of specimens from additional localities globally. These represent a much smaller percentage of the material than North American and European specimens. The collections examined are extensive and were surveyed as part of a larger study on bioerosion and as an examination of depositional environments, and only particular subsets of the available material are appropriate for searching for and describing *Osedax* borings. Disarticulated specimens from oxygenated depositional environments yielded the highest degree of both *Osspecus* and other forms of bioerosion. A subset of the total material deemed to have promising bioerosion resembling *Osspecus* at the surface was selected for CT scanning. See individual museum databases for available lists of specimens and marine reptile taxa within these collections.

### (b) CT scanning

Twenty specimens with surface traces resembling *Osspecus* as seen from the exterior were CT scanned (table 1). These scans were of specimens with varying degrees of pitting and weathering, and sourced from a number of rock units representing a broad range of depositional environments (table 1). Scans of UK material were carried out at NHMUK with the Nikon Metrology HMX ST 225 micro-CT scanner, while specimens from the FMNH were scanned with the University of Chicago PaleoCT (RRID:SCR_024763). Belgian specimens were scanned at IRSNB on the RX Solutions EasyTom150 micro-CT scanner. Scan settings and filters were variable depending on the density, size and preservation of individual fossils. See the documents with details of scan parameters provided alongside the CT scan image stacks in the data supplement (https://www.morphosource.org/projects/000582794?locale=en). These machines provide sufficient resolution for the identification of mm to cm scale *Osedax* borings. The scans were processed, and the borings segmented and three-dimensionally reconstructed using the software Avizo (FEI Visualization Science Group; https://www.thermofisher.com). Other specimens bearing potential bioerosion with a superficial resemblance to *Osedax* borings were not scanned, as these instances were determined to be too indefinite or unlikely to be related to *Osspecus* bioerosion.

**Table 1.** Specimens scanned due to a surface identification of possible *Osedax* bioerosion. Specimens determined to have *Osedax* borings are marked in bold and with an asterisk.

specimen number	specimen	stratigraphic age	geographic locality
OUMNH.PAL-J12321	*Muraenosaurus* vertebra	Jurassic (Kimmeridgian)	United Kingdom
OUMNH.PAL-J12340.2	plesiosaur vertebra	Jurassic (Kimmeridgian)	United Kingdom
CAMSM TN5563.1-4	pliosaur: two vertebrae and two jaw elements	Jurassic (Callovian–Oxfordian)	United Kingdom
CAMSM J.29712	*Plesiosaurus* ischium	Jurassic (Kimmeridgian)	United Kingdom
NHMUK PVR 275	*Ichthyosaurus ‘?trigonus’* left humerus	Jurassic (Kimmeridgian)	United Kingdom
NHMUK PVR 6680	*Plesiosaurus* element	Cretaceous (Berriasian–Albian)	United Kingdom
NHMUK 40450	*Plesiosaurus* vertebra	Cretaceous (Aptian)	United Kingdom
NHMUK PVR 36385	*Ichthyosaurus campylodon* paddle bone	Cretaceous (Albian)	United Kingdom
NHMUK 36150a	*Plesiosaurus* bernardi vertebra	Cretaceous (Albian)	United Kingdom
NHMUK PVR 1264	*Polyptychodon interruptus*' teeth and jaw	Cretaceous (Cenomanian–Maastrichtian)	United Kingdom
*NHMUK PVR 35103	***‘Polyptychodon interruptus***' **tooth**	**Cretaceous (Cenomanian)**	**United Kingdom**
NHMUK PVR 2579	elasmosaur paddle bone	Cretaceous (Maastrichtian)	Antarctica
*NHMUK PVR 5869	**elasmosaur vertebra**	**Cretaceous (Maastrichtian)**	**United States of America**
FMNH P 12013	*?Polycotylus* fragmentary elements	Cretaceous (Coniacian–Campanian)	United States of America
*FMNH PR 187	***Polycotylus latipinnis* partial skeleton**	**Cretaceous (Campanian)**	**United States of America**
FMNH PR 56	elasmosaur partial skeleton	Cretaceous (Maastrichtian)	United States of America
IRSNB R 299	*Mosasaurus lemonnieri* partial skeleton	Cretaceous (Maastrichtian)	Belgium
*IRSNB R 369	***Mosasaurus lemonnieri* partial skeleton**	**Cretaceous (Maastrichtian)**	**Belgium**
*IRSNB R 370	***Mosasaurus lemonnieri* partial skeleton**	**Cretaceous (Maastrichtian)**	**Belgium**
IRSNB R 400	*Mosasaurus lemonnieri* partial skeleton	Cretaceous (Maastrichtian)	Belgium

## 3. Results

**Figure 1.**
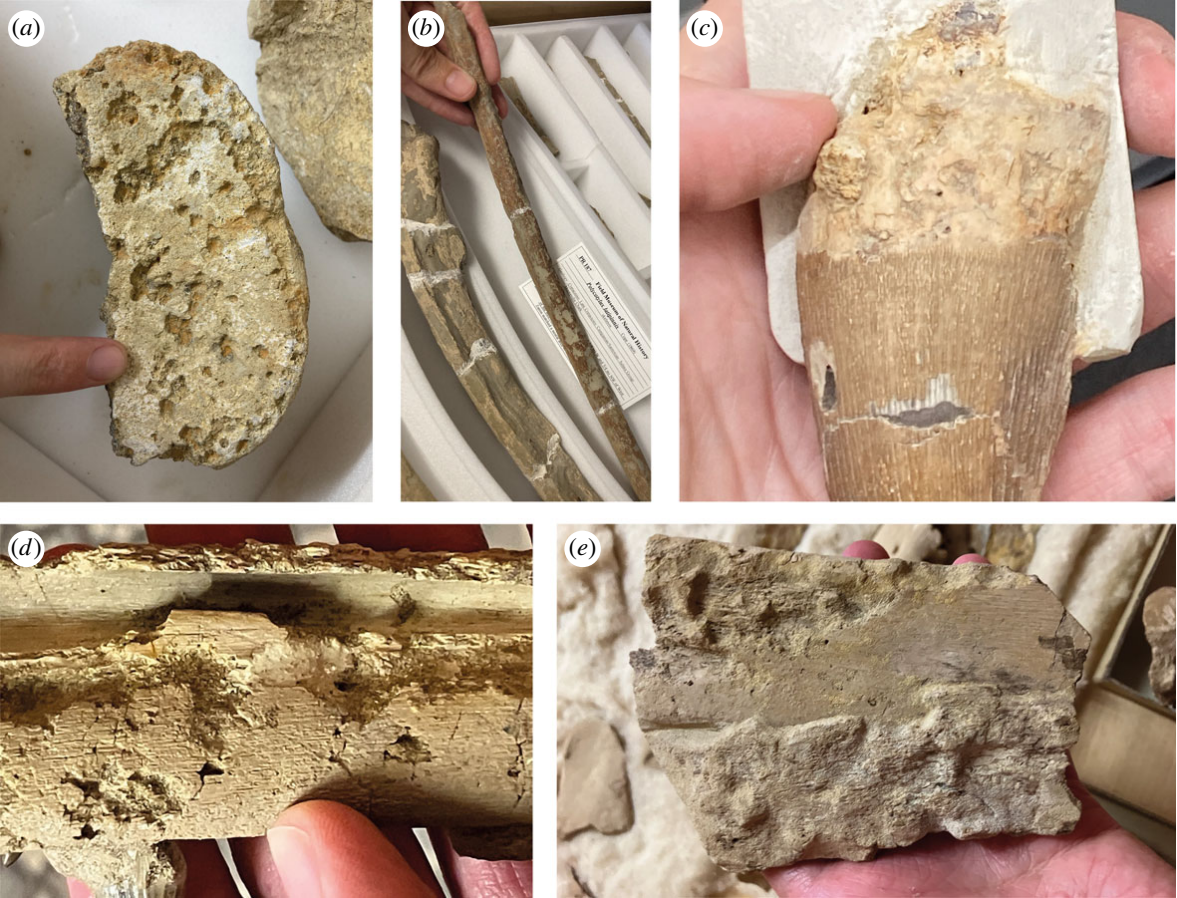
Surface bioerosion of the five specimens with *Osspecus* traces. (*a*) Pitting on the vertebra from NHMUK PVR 5869. (*b*) Heavy pitting on the rib of FMNH PR 187. (*c*) NMHUK PVR 35103 showing ‘pinhole’ type bioerosion on the tooth rooth that represents the entrances to *Osspecus* chambers. (*d*) Scanned jaw piece from IRSNB R 369 showing partially collapsed chambers. (*e*) Scanned jaw piece from IRSNB R 370 showing ‘pinholes’ and pitting.

The most promising scans were of specimens sourced from the UK, Belgium and the southeastern United States, housed in NHMUK, IRSNB and FMNH. See the data supplement for CT scan image stacks of the relevant specimens (https://www.morphosource.org/projects/000582794?locale=en). In all cases the bones with the highest degree of bioerosion combined with the best-preserved bioerosion were selected for scanning. Five specimens were determined to have *Osspecus*: an indeterminate pliosaurid plesiosaur tooth (NHMUK PVR 35103), an indeterminate elasmosaurian plesiosaur partial skeleton (NHMUK PVR 5869), a partial skeleton of the polycotylid plesiosaur *Polycotylus latipinnis* (FMNH PR 187), and two fragmentary mosasaur skeletons (IRSNB R 369 and IRSNB R 370) (figure 1, table 1). These descriptions add five additional instances to the known marine reptile specimens with *Osspecus* from the Mesozoic, including the first identification of *Osspecus* in mosasaurs. See supplementary scan data for exact surface and internal images of the bioerosion and its location and extent on the bones, and table 2 for relative sizes and geometries of the borings. The ranges of width and depth of the *Osspecus* chambers are in line with the range of sizes in chambers created by present-day *Osedax* [15].

**Table 2.** Elements of specimens which exhibit *Osspecus* when scanned, and the relative dimensions and characteristics of the borings. Note that there is only one example of type 2 and 3 borings.

boring type	characteristics	depth range (mm)	diameter range (mm)	specimen number
type 1	thick radial branches form main part of chamber	1–5	0.5–3	NHMUK PVR 35103
type 2	deep lobate chamber	2.7	0.6	NHMUK PVR 35103
type 3	filamentous radial branches off central deep chamber	1.5	1	NHMUK PVR 35103
type 4	relatively larger, shallow, hemispherical chamber with filamentous radial branches	1.5–3.5	1.5–4.5	NHMUK PVR 5869
type 5	relatively smaller, central chamber with radial branching	0.5–1.5	1–3	IRSNB R 369; IRSNB R 370; FMNH PR 187

### (a) NHMUK PVR 35103

**Figure 2.**
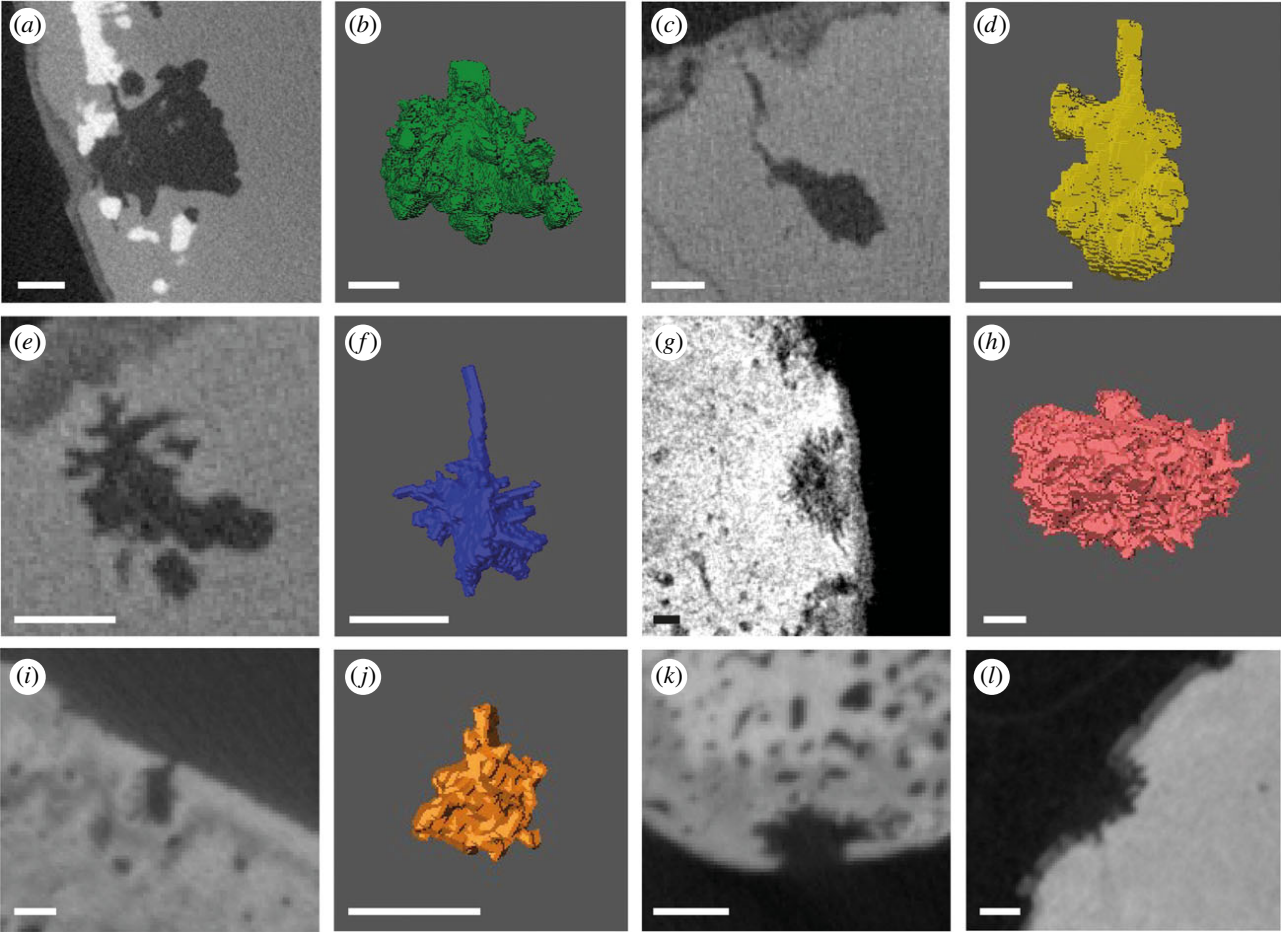
Five boring morphotypes exhibited in marine reptile bones. (*a*) Type 1 boring in NHMUK PVR 35103, with a radial lobate branching structure. (*b*) Three-dimensional reconstruction of type 1 boring. (*c*) Type 2 boring NHMUK PVR 35103, with a deep lobate chamber. (*d*) Three-dimensional reconstruction of type 2 boring. (*e*) Type 3 boring in NHMUK PVR 35103, with a central chamber and filamentous branches. (*f*) Three-dimensional reconstruction of type 3 boring. (*g*) Type 4 boring in NHMUK PVR 5869, with a hemispherical chamber and ‘frayed’ branching edges. (*h*) Three-dimensional reconstruction of type 4 boring. (*i*) Type 5 small-scale radial boring in IRSNB R 370. (*j*) three-dimensional reconstruction of type 5 boring. (*k*) Indeterminate, possibly type 5 boring in IRSNB R 369. (*l*) Indeterminate, possible type 5 boring in FMNH PR 187. Scale bars are 1 mm.

This single tooth comes from the Cenomanian Lower Chalk of the UK. Most borings are on the root and a few clusters of borings on the crown, forming ‘pinholes’ on the surface that represent the entrances to the borings. *Osspecus* chambers of at least three distinct morphologies extend beneath these visible apertures (figure 2), one with thicker radial branches (type 1), one with a deep lobate chamber (type 2), and one with a central chamber and more filamentous branches (type 3), indicating that there might have been multiple species of *Osedax* exploiting the dentine of this tooth (table 2).

### (b) NHMUK PVR 5869

This specimen is from the Campanian Marlbrook Marl of Arkansas. One vertebra was scanned from this partial skeleton, because only two vertebrae have obvious boreholes. These vertebrae shows heavy bioerosion on one surface (figure 1). It is not clear why *Osedax* targeted these particular vertebrae, but most of the elements from this partial skeleton exhibit surface traces that may be sea urchin grazing, so it is possible that *Osedax* arrived quite a while after the animal's death when much of the skeleton had already been grazed and buried. NHMUK PVR 5869 exhibits an additional borehole morphology (type 4), consisting of a hemispherical chamber with thin radial branches, creating a frayed appearance at the edges of the chamber (figure 2, table 2). Many of these chambers are visible at the surface as collapsed pits, while others show only from the surface as ‘pinholes’ representing the aperture.

### (c) FMNH PR 187

This specimen is from the Campanian Mooreville Chalk of Alabama. Two ribs and a partial vertebra were scanned from this partial skeleton (figure 1). The highest degree of bioerosion on this specimen is on the ribs, and to a lesser degree on vertebrae, but additional borings are seen on the other elements as well. It is likely that ribs and neural arches have a tendency to project above the sediment surface and remain exposed for longer relative to the rest of the skeleton. The borings are more weathered and less definitive in this specimen, showing mainly as pits at the surface that are generally fully or partially collapsed, but are also likely to be of type 5 based on their internal shape (figure 2).

### (d) IRSNB R 369 and IRSNB R 370

The two mosasaur specimens come from the Maastrichtian chalk of the Mons Basin in Belgium. A rib and mandible from IRSNB R 369, and a mandible from IRSNB R 370 were scanned (figure 1). For both of these partial skeletons, the degree of bioerosion is moderate, and on the mandibles it is generally the case that one side of the bone has a higher degree of bioerosion than the other, serving as a ‘this-way-up’ taphonomic indicator. The rib of IRSNB R 369 is more heavily pitted on all sides than the other specimens. These have very small-scale borings relative to the other boring types, with a deep chamber and radial filamentous branches (type 5) (table 2). IRSNB R 370 has uncollapsed borings, with a thin layer of bone still covering some of the chambers, and IRSNB R369 has borings that are mostly collapsed, with the chamber exposed to the surface, of the same type (figure 2).

## 4. Discussion

### (a) Depositional environments and sampling

The new Cretaceous evidence for *Osspecus* described here comes from specimens that are all sourced from relatively shallow-water, well-oxygenated depositional environments, which would have been abundant in the Cretaceous, but the range of depths that *Osedax* occupied during this time is not currently known. Fossil record bias in the rock record leads to an over-representation of shallow-marine environments [23], and the presence of *Osedax* in deeper marine environments in the Cretaceous has not yet been confirmed.

The number of fossil marine reptiles with *Osspecus* relative to the total number of marine reptiles in collections is unlikely to be a realistic representation of the true percentage of marine reptile carcasses colonized by *Osedax* prior to burial and fossilization. This preservational bias is exacerbated by bioeroders which degrade bone and reduce preservation potential and which are more present in oxygenated environments. In the case of bone-eating worm bioerosion this degredation and potential decrease in preservation potential is termed the ‘*Osedax*’ effect [19]. It is not possible to quantify the full influence of this effect on preservation in the fossil record due to the inherent bias, but it is likely to have affected the degree of preservation of many marine reptile fossils and potentially the volume of available material with *Osspecus.* Much of the marine reptile material from collections that was examined during the course of this study, particularly the Jurassic material, is also very well preserved and representative of lower oxygen environments where invertebrates not known to tolerate anoxia, such as *Osedax*, would not be expected. Some of the Jurassic specimens show other forms of invertebrate bioerosion or unidentified pitting and boring in instances where the environment was more oxygenated [18], but still exhibit no instances of definitive *Osedax* borings. It stands to reason, given that *Osedax* has not been found in anoxic environments today, that this metabolic and ecological preference may have also existed in Mesozoic members of this genus, and that Jurassic environments with too little oxygen on the ocean floor to support other invertebrate bioeroders would have also excluded *Osedax*. Conversely, where other forms of invertebrate bioerosion are present and the environment appears to have been better oxygenated, there is no reason why *Osedax* should be absent as a result of sampling bias or taphonomic factors. These observations could be interpreted as indicating that *Osedax* was genuinely absent in the Jurassic, but caution is required. The relative scarcity of well-oxygenated Jurassic marine environments available for sampling may have led to a misrepresentation of the frequency and prevalence of *Osedax* in marine reptiles generally, and these factors are not well-understood. Oxygen is not the only driver of taphonomic patterns, and *Osedax* borings are unlikely to be preserved in bones that are not buried halfway through decomposition, as the *Osedax* themselves exert a taphonomic bias. Additional specimens will be required to determine whether *Osedax* was genuinely absent in the Jurassic.

### (b) Distribution, origin and diversity of *Osedax* in the Cretaceous

**Figure 3.**
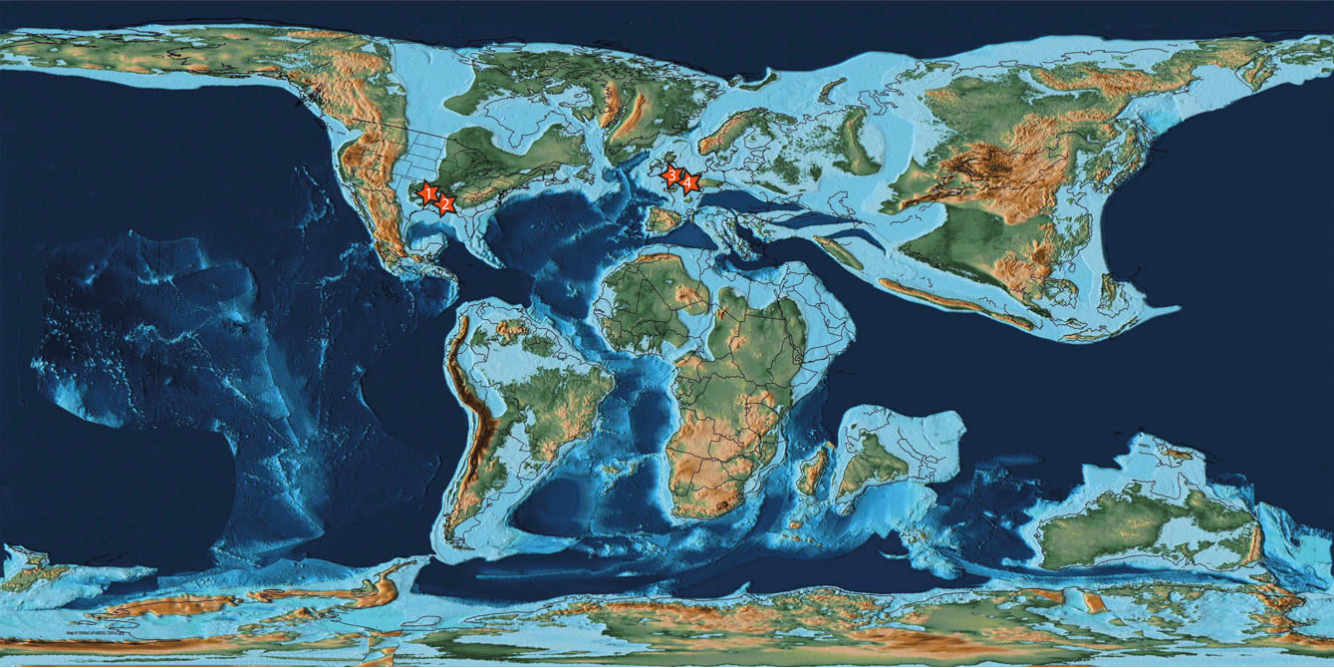
A PALEOMAP PaleoAtlas [24] map showing the arrangement of continents and deep and shallow marine environments in the Campanian. Stars indicate fossil occurrences of *Osedax* newly described here*.* The numbers represent: (1) NHMUK PVR 5869; (2) FMNH PR 187; (3) NHMUK PVR 35103; (4) IRSNB R 369 and IRSNB R 370.

Mesozoic instances of *Osspecus* had previously been reported only from the Albian and Cenomanian of the UK [13]. Here we have identified an additional UK example, alongside the first occurrences from North America, and the first occurrences in mosasaurs (from Belgium). The US specimens are from the south-eastern Western Interior Seaway and the connected Gulf Coastal Plain, adjacent to the western margin of the expanding North Atlantic Ocean basin (figure 3). This places *Osedax* in western Europe by the late Early Cretaceous, in line with previous descriptions of *Osspecus* from the mid-Cretaceous of the UK [13], and in North America by the Late Cretaceous.

*Osedax* dispersal relies on distribution of a larval stage in the water column [25]. These larvae may be carried by ocean currents, settling and spawning again where vertebrate remains are available [25]. One consequence of marine larval dispersal is that the phylogenetic relationships of extant species of *Osedax* are not well reflected in their present-day geographic distribution [26]. The duration of larval survival in the water column is not well understood, but, in order to reproduce, the larvae must find bones on which to settle. It is likely that the survival potential of larvae differs between species, with factors such as water temperature and depth, as well as egg size, playing a role [27]. Larvae of *Osedax japonicus* have been kept alive in tanks for ten days before settlement was induced by adding pieces of whale bone to the tanks [28]. This provides a minimum estimate for survival time of larvae for at least this extant species [28]. However, laboratory tanks are a very different environment to the open ocean that is the natural habitat for *Osedax*, and the species present in the Cretaceous may have differed biologically from living species. Putting aside potential constraints, an organism that can disperse as larvae travelling via ocean currents could have achieved a widespread or even global distribution on a geologically ‘instantaneous’ timescale. This seems even more feasible in the marine environments of the Late Cretaceous, when more of the Earth's surface was covered by water than in the present day [24]. Moreover, the Late Cretaceous was characterized by an increase in ocean circulation and a greater water exchange between the North and South Atlantic as the Atlantic Ocean basin continued to expand, as well as extensive current flow between major ocean basins [27,28]. The presence of *Osspecus* in disparate regions of the North Atlantic Ocean basin during the Late Cretaceous suggests that it may also have been distributed throughout the entire Atlantic Ocean basin, or possibly even globally, by this time. Additional data points from the Cretaceous of the Southern Hemisphere, and from the margins of other major ocean basins, will be needed to determine the full extent of the distribution of *Osedax* during the Cretaceous.

Studies of modern whale bone demonstrate that a given species of *Osedax* tends to produce a consistent borehole morphotype within the same bone and bone type [14,15]. The other examples identified here that show one morphotype per individual skeleton are consistent with these findings, and multiple skeletal elements from the same specimen continue to show the same consistent borehole morphology, suggesting that one species is unlikely to make distinctly different borehole morphotypes in the same skeleton regardless of ontogenetic stage or time of settlement. Variation in borehole morphology made by members of the same species has been previously shown as attributable to porosity and density of bone [14], but where distinctive geometries are present, as in this study, the most likely explanation is that different species were present. Certain fossil borings described here bear a resemblance to types of *Osedax* borings that have been previously described. For instance, our type 1 boring bears a resemblance to borings in an Oligocene whale tooth [20], our type 5 borings have some features shared with modern *Osedax mucofloris* borings [14], our type 4 with modern *Osedax japonicus* borings, and our type 3 with modern borings from an undescribed species termed ‘yellow palp’ [15]. These similarities may not be taxonomically or phylogenetically informative, as there is no evidence to show that particular sub-groups of *Osedax* create similar boring types rather than having borings with convergent morphologies, but these similarities nonetheless strengthen the evidence for these instances of *Osspecus* being created by *Osedax*. The presence of three boring morphologies in the pliosaur tooth from the Cenomanian of the UK, and two additional borehole morphotypes in the North American and Belgian specimens, suggests that the radiation of *Osedax* was well underway during the Late Cretaceous, with multiple species colonizing marine reptile bones on the ocean floor by the Cenomanian. That multiple boring morphotypes are present within the same skeletal element and bone type (a single tooth) further supports this conclusion.

Molecular clock estimates that have suggested an origin of *Osedax* in the mid-Cretaceous have calibrated phylogenies based on the evolutionary rates of deep-sea annelids [11] or on the earliest known fossil occurrences of *Osedax* [12], and have placed the subsequent radiation of this clade later in the Cretaceous or (at the latest) in the earliest Eocene [11,12]. Fossil evidence has already shown that *Osedax* was present in the mid-Cretaceous and did not originate and diversify alongside cetaceans [13]. The diversity of boring morphologies that we present here in Cretaceous examples further indicates that *Osedax* either experienced an adaptive radiation or that it may have originated earlier than previously thought, and that additional stratigraphically older fossil examples are likely to be found in the future.

## 5. Conclusion and prospects

We describe five new instances of bioerosion produced by the bone-eating worm *Osedax* in Late Cretaceous marine reptiles. Two of these occurrences come from Campanian deposits in the southeast of the United States and expand the known geographic range of *Osedax* in the Mesozoic to the western margin of the North Atlantic Ocean. Five borehole morphotypes represented in these two specimens and newly described specimens from the Cenomanian of the UK and Maastrichtian of Belgium suggest a higher species diversity of *Osedax* at this time than previously realized.

Extending the geographical range of *Osedax* in the fossil record may help to determine at which point in time this clade became globally distributed. Identifying additional boring morphologies and using these as a proxy for diversity will aid in tracking the diversification of this clade, and help to calibrate its potential time of origination. There is a significant amount of marine reptile material with a global distribution in the Jurassic and Cretaceous that has not yet been examined, and with further work we predict that further instances of *Osspecus* in fossil bone are likely to be found earlier in the Mesozoic and outside of the Atlantic Ocean basin.

## Data Availability

CT scan image stacks for the scanned elements from specimens determined to have *Osedax* borings and their associated scan parameter files are stored on MorphoSource: https://www.morphosource.org/projects/000582794?locale=en. Individual scan parameter files are uploaded with the relevant scans.
